# Simulated Interactive Research Experiments as Educational Tools for Advanced Science

**DOI:** 10.1038/srep14108

**Published:** 2015-09-15

**Authors:** Mathias Tomandl, Thomas Mieling, Christiane M. Losert-Valiente Kroon, Martin Hopf, Markus Arndt

**Affiliations:** 1 University of Vienna, Faculty of Physics, VCQ, QuNaBioS, Boltzmanngasse 5, 1090 Vienna, Austria; 2 University of Vienna, Faculty of Physics, AECCP, Porzellangasse 4, 1090 Vienna, Austria

## Abstract

Experimental research has become complex and thus a challenge to science education. Only very few students can typically be trained on advanced scientific equipment. It is therefore important to find new tools that allow all students to acquire laboratory skills individually and independent of where they are located. In a design-based research process we have investigated the feasibility of using a virtual laboratory as a photo-realistic and scientifically valid representation of advanced scientific infrastructure to teach modern experimental science, here, molecular quantum optics. We found a concept based on three educational principles that allows undergraduate students to become acquainted with procedures and concepts of a modern research field. We find a significant increase in student understanding using our Simulated Interactive Research Experiment (*SiReX*), by evaluating the learning outcomes with semi-structured interviews in a pre/post design. This suggests that this concept of an educational tool can be generalized to disseminate findings in other fields.

Laboratory settings have often been recommended for training students in practical applications of science^1^,^2^, but appropriate learning environments for modern research are rare. Today, science is highly interconnected, often multidisciplinary and complex^3^; it therefore requires adequate new learning tools. While the expenses for laboratory infrastructure often prevent its use for training purposes, computational technologies now allow the realization of virtual laboratories that can serve learning equally well^4^. These tools can also provide access to experiments that would be too hazardous to be used in a classroom^5^. How to create and use such learning tools is a growing field of educational research^6^,^7^,^8^. Here, we present the concept of Simulated Interactive Research Experiments (*SiReX*) that have been developed in a design-based research^9^,^10^. *SiReX* combine photo-realistic real-time simulations (Fig. 1) with a comprehensive series of cognitive tools^11^. We demonstrate the feasibility of this approach for the example of quantum diffraction and interferometry with large molecules. This realization of the concept has been evaluated with regards to learning outcomes of undergraduate students.

## The SiReX concept - A virtual laboratory for advanced research

Matter-wave physics has already entered numerous text books for high schools and undergraduate teaching^12^. But written text can never provide the touch and feel of a genuine experiment and therefore often not even trigger the question why and how science works. In reply to this gap we demonstrate the *SiReX* concept for the specific example of two real-world research instruments. Nanograting diffraction of molecules allows to observe the wave-particle duality of complex matter in real-time^13^. The Kapitza-Dirac-Talbot-Lau interferometer (KDTLI) allows to explore molecule interferometry up to masses in excess of 10.000 atomic mass units (amu)^14^,^15^.

The aim of the *SiReX* concept is to train students in practical applications of modern scientific research and to provide the required background information simultaneously. Hence, it needs to show scientifically valid outcomes, mimic real interaction with the laboratory equipment and be simple enough to be understood by undergraduate students. *SiReX*s shall guide students through a complex modern infrastructure and in passing teach a comprehensive set of subjects, here from thermodynamics over mass spectrometry, to advanced elements of optics and quantum optics. This approach should reveal the interconnectedness of different concepts of experimental physics and allow training students in applying their knowledge in a real-world environment. This multi-topic approach differs from many earlier interactive learning tools for quantum physics^16^,^17^,^18^ that refer to one specific concept or phenomenon.

**Figure 2:**
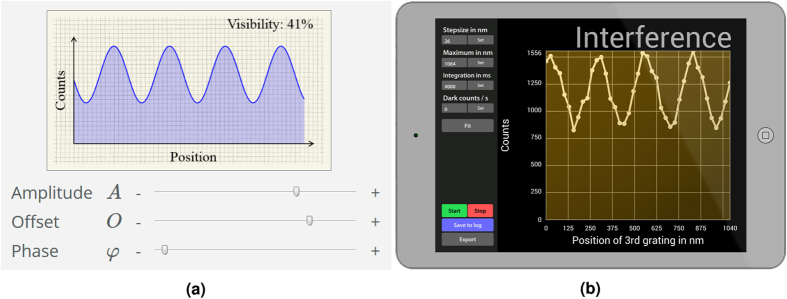
Multiple representations of science in *SiReX LE*. (a) Interactive illustrations in the *SiReX Knowledge Base* visualize the mathematical background and concepts of molecular matter-wave experiments. They are limited to a handful of parameters and are free of noise. (**b**) In the *SiReX Laboratory* students can control a wide range of parameters on a virtual tablet to conduct experiments with realistic noise and restrictions of the real-world setup.

To fulfill these goals the *SiReX Learning Environment* (*SiReX LE*) was created in order to realize and test the concept. It is freely available online^19^ and consists of two parts: the *SiReX Knowledge Base* and the *SiReX Laboratory* (Table 1). The *SiReX LE* is based on three principles from educational research: (i) to guide learners dynamically, adapted to their progress^20^; (ii) to support students with cognitive tools^11^; and (iii) to provide multiple representations^21^,^22^ for connecting concepts and with phenomenological applications (Fig. 2).

### The SiReX Knowledge Base

Provides the scientific background via explanatory texts, schematic illustrations and conceptual applets. The information focuses on basic theory and qualitative relations between the relevant parameters. It is complemented by simplified graphical illustrations, interactive function graphs and applets which assist in visualizing the scientific models (Fig. 2a), similar to Physlet^23^, PhET^24^ and others^18^,^25^,^26^. The visualization of fundamental concepts in a restricted parameter space helps students to prepare for their experimental assignments in the *SiReX Laboratory*. Additional quantitative information can be optionally accessed. Questionnaires allow the students to verify their understanding before they are guided to their next experimental task in the virtual research laboratory.

### The SiReX Laboratory

**Figure 3:**
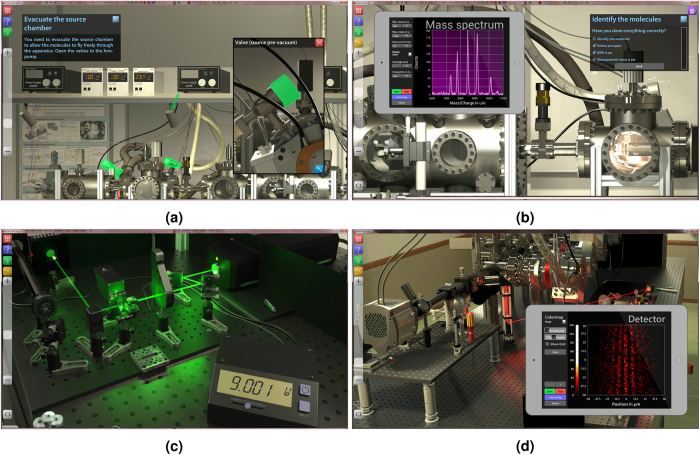
Examples of student actions in the *SiReX Laboratory*. (**a**) Evacuating the vacuum chambers; for easier orientation, the most relevant parts are highlighted in color; message boxes support the learner with instructions; (**b**) Recording and analyzing a mass spectrum to characterize the molecular signal; (**c**) Adjusting the laser power using polarization optics. (**d**) Measuring the full two dimensional interference pattern of molecules.

Enables the students to apply the acquired concepts in game-like experimental challenges. It provides photo-realistic 3D lab visualizations, touch-compatible gesture control and audio-visual feedback (Fig. 3). An on-demand information system describes details of the experimental equipment and visually highlights the relevant parts of the setup, to avoid cognitive overload. The simulation provides a dynamic guidance that gradually fades away as the user proceeds^20^. Thereby, additional interfaces and more complex challenges are gradually made accessible to help students finding their way from setting up a first experiment to processing the data. They are confronted with state-of-the-art lab technology such as molecular beam physics, vacuum technology, nanopositioning, vibration isolation, high-power lasers, fluorescence microscopy and mass spectrometry, in the case of our two specific demonstrations.

### Two paradigmatic examples

#### Single-molecule imaging of quantum interference

Observing interference patterns of massive particles in double-slit experiments is a widely discussed demonstration of their quantum nature. The build-up of such an interference pattern by individual particles has been observed with electrons, neutrons, atoms and more recently with complex molecules^13^. *SiReX* allows the students to conduct this experiment in the virtual laboratory and observe single particles arriving at the detector surface one by one. With a laser-micro-evaporation source the students produce a beam of single molecules and position nanometer-thick gratings into the beam. Using fluorescence microscopy they detect individual positions of molecules and reveal the build-up of a deterministic ensemble interference pattern from stochastically arriving single molecules.

#### Studying collisional decoherence

In a more advanced example students can observe collisional decoherence in a near-field interferometer for complex molecules. One explanation, why matter-wave interference is not observed in every day life is that even minimal interactions with the environment shift the quantum phases randomly. While systematically raising the pressure in the vacuum chamber the users make interference scans. Collisions with residual gas molecules localize the molecular center-of-mass wave function leading to a reduced visibility of the interference pattern. Thereby students can explore the transition between genuine quantum observations and classical phenomena. Such an experiment could not be provided as a physical educational tool for students. The *SiReX LE* removes that restriction and allows learning at such high-tech equipment for everyone.

## Discussion

### Evaluation with qualitative content analysis

We conducted and analyzed 59 interviews, with physics major students (*n* = 23) and students from the physics teacher accreditation program (*n* = 7) during the development of our *SiReX*. The evaluation was done at four different stages in the development process. We found good acceptance among the students for the dual structure of the *SiReX LE* which separates the concept learning from the laboratory experience. In the first two evaluation cycles (*n* = 7) the students were working with the *SiReX Laboratory* with guidance by the instructor. These interventions were video recorded in a think-aloud format. The students were asked to speak out what they were thinking while operating the virtual lab. The purpose of this intervention was to define a learning path that meets the students’ level of understanding and links to their prior knowledge. Based on this analysis the *SiReX Knowledge Base* was developed. The second two cycles (*n* = 11 and *n* = 12) were done with semi-structured interviews in a pre/post scheme using the now established *SiReX Knowledge Base* to provide instructions and guidance.

Before interacting with the *SiReX* environment most students had difficulties imagining how all the phenomena, they had heard about in lectures, could be observed in the real world. This observation even holds for students who had worked through a script about the molecule experiment up-front.

**Figure 4:**
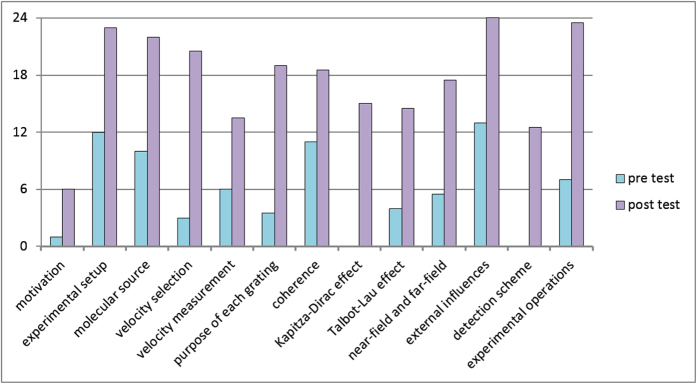
Results of the *SiReX*-only evaluation: The graph shows the total points of twelve students summed up for each item before and after the use of *SiReX*. In total, the students scored three times higher after using *SiReX* compared to the first interview. While they started together from 76 points in the pre-test they scored 227 points in the post-test.

The *SiReX LE* improved the student’s understanding in all evaluated learning items by a factor of three on average (Fig. 4). An adequate improvement was achieved in technical topics like the velocity measurement or mass-spectrometry. A strong improvement was achieved in modules that could be connected to prior knowledge such as molecular beam sources or gravitational velocity selection. In entirely new fields like near-field interference effects, the students strongly improved their knowledge after their interaction with *SiReX*. The students perceived the virtual lab as realistic, detailed and entertaining.

Even though additional quantitative research needs to be conducted on learning outcomes in large scale settings our results indicate that *SiReX* can be useful for interdisciplinary fields of equipment intensive research, also at the interface between physics, chemistry, and biology in the future.

## Methods

### Design based development

With design-based research^9^,^10^ as a systematic but flexible method, we started with a development plan that evolved iteratively during the research process on the *SiReX Learning Environment* through design, analysis and adaptive development. This allowed us to refine the methods by valuing principles that improved the learning practice. We used individual ‘think-aloud’ and semi-structured interviews during different phases of the development. Some tested the effect of the *virtual lab alone*; some a *hybrid setting* where students interacted first with the virtual and only then with the real-world laboratory. Our outcome-oriented research was conducted for the example of the Kapitza-Dirac-Talbot-Lau interferometer. This is an advanced experiment of modern quantum optics, which is rather thought on the master level. The concepts derived from this educational research were then translated to a second *SiReX* on *Molecular Diffraction*, which has fewer elements, is conceptually simpler and in practice often used in modern physics textbooks, even already in secondary education. Based on the evaluations and feedback from the educators the *SiReX* environment was iteratively redesigned and optimized to be self explanatory and simple to use. Photo-realistic visualization combined with accurate simulation of noise and signals allow an authentic look and feel similar to *Interactive Screen Experiments*^27^ or *Remotely Controlled Laboratories*^28^,^29^. In comparison to these, the *SiReX Laboratory* provides a high degree of interactivity and the computational model contains more than 100 user accessible parameters. Also, SiReXs can serve many students simultaneously, which makes them compatible with lectures, outreach activities, high-school teaching^30^ or web-based distance learning.

### Evaluation details

The evaluation was integrated into a lab course on experimental quantum optics at the University of Vienna and focused on 13 topics concerning the molecular quantum interference experiment. They were asked about the motivation to conduct such experiments (i), to explain the experimental setup (ii), the characteristics of the molecular source (iii), coherence considerations (iv), the velocity selection (v), the velocity measurement (vi), the purpose of each of the three grating of the setup (vii), the Kapitza-Dirac effect (viii), the Talbot-Lau effect (ix), differences between the optical near-field and far-field (x), possible implications from external influence (xi), the detection process (xii) and to list the most relevant operations for conducting the experiments (xiii). The interviews were transcribed and evaluated based on predefined categories in a *qualitative content analysis*^31^. The answers were rated on a three-level scale according to the criteria for each individual topic. An answer was valued 2 points if it covered all or most of the requested information correctly; 1 point was given if the answer contained relevant information but either was not specific or comprehensive enough; 0 points were assigned if the given answer did not meet the minimum requirements. The examination was done by two evaluators and tested for inter-rater reliability with a Cohen’s Kappa >0.9^32^. Half points could be assigned if the two evaluators did not rate the answer identically.

### Scientifically valid simulations

The interaction and outcomes of the virtual laboratory are generated with a complex numerical model that simulates the entire experimental setup, from a simple vacuum valve to the interaction of molecules with a standing light wave. The system is highly interdependent, e.g. the voltage set at the power supply changes the temperature of the source that affects the evaporation rate of the molecules dependent of their vapor pressure; a higher evaporation rate affects the pressure in the vacuum chamber and therefore the transmission to the detector as well as the visibility of the interference fringes. The velocity spectrum of the molecular beam depends of the position of several beam delimiters, it shows instantaneous feedback at the real experiment. The dynamic computation of such physical processes with real-time feedback could only be realized with an asynchronous software architecture that uses not-blocking algorithms to avoid freezing of the user interface while calculations are done. In some cases heavy calculations are iteratively performed with increasing precision, providing rough values from the start. The errors of the simulation due to down-sized integration parameters and simplified models are kept below the noise limit and is therefore not noticeable for the user. The high complexity and interdependence of parameters leads to a nonlinear behavior of the simulation. This intends to foster students creativity at identifying the dependencies.

## Additional Information

**Accession codes:** The result of this research can be freely accessed through the following permanent link: http://interactive.quantumnano.at/. The current implementation is based on Adobe Flash^©^, but the concept can be freely migrated to HTML5 or any other Browser code.

**How to cite this article**: Tomandl, M. *et al.* Simulated Interactive Research Experiments as Educational Tools for Advanced Science. *Sci. Rep.* **5**, 14108; doi: 10.1038/srep14108 (2015).

## Figures and Tables

**Figure 1:**
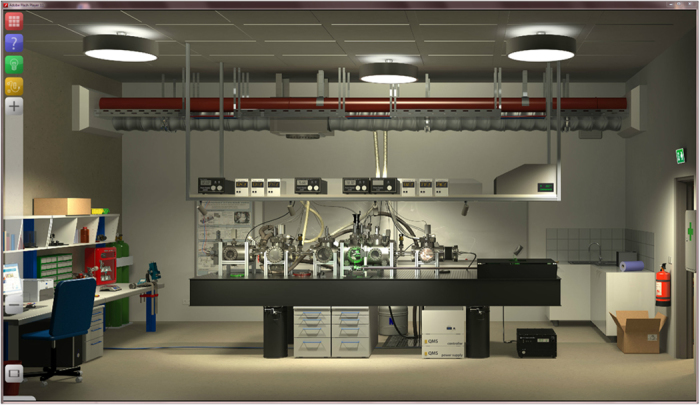
The *SiReX Laboratory*: A photo-realistic 3D representation of an existing laboratory allows to explore molecular quantum optics.

**Table 1:** Two parts of the SiReX Learning Environment.

SiReX Knowledge Base	SiReX Laboratory
Background information	Experimental interaction
Simplified conceptual models	Complex phenomenological models
Not more than 3 parameters	More than 100 parameters
Schematics	Photo-realistic 3D graphics
Interaction with sliders and buttons	Control of virtual equipment: lasers, optics, electronics, etc.
